# Role of S100 Proteins in Colorectal Carcinogenesis

**DOI:** 10.1155/2016/2632703

**Published:** 2016-01-06

**Authors:** Paula Moravkova, Darina Kohoutova, Stanislav Rejchrt, Jiri Cyrany, Jan Bures

**Affiliations:** ^1^2nd Department of Internal Medicine-Gastroenterology, Charles University in Praha, Faculty of Medicine at Hradec Kralove, University Teaching Hospital, Sokolska 581, 500 05 Hradec Kralove, Czech Republic; ^2^Division of Surgery and Interventional Science, University College London, 67-73 Riding House Street, London W1W 7EJ, UK

## Abstract

The family of S100 proteins represents 25 relatively small (9–13 kD) calcium binding proteins. These proteins possess a broad spectrum of important intracellular and extracellular functions. Colorectal cancer is the third most common cancer in men (after lung and prostate cancer) and the second most frequent cancer in women (after breast cancer) worldwide. S100 proteins are involved in the colorectal carcinogenesis through different mechanisms: they enable proliferation, invasion, and migration of the tumour cells; furthermore, S100 proteins increase angiogenesis and activate NF-*κβ* signaling pathway, which plays a key role in the molecular pathogenesis especially of colitis-associated carcinoma. The expression of S100 proteins in the cancerous tissue and serum levels of S100 proteins might be used as a precise diagnostic and prognostic marker in patients with suspected or already diagnosed colorectal neoplasia. Possibly, in the future, S100 proteins will be a therapeutic target for tailored anticancer therapy.

## 1. Introduction

The family of S100 proteins represents a total of at least 25 relatively small (9–13 kD) Ca^2+^ (calcium) binding proteins. They are expressed exclusively in the vertebrates and their function gets changed in a response to the changing calcium concentration. Their name is derived from the fact that many members are soluble in 100% ammonium sulfate at the neutral pH [1]. S100 proteins have a broad range of functions; they play a role in the regulation of cell proliferation, differentiation, apoptosis, energy metabolism, cellular signaling, and Ca^2+^ homeostasis. Extracellulary, S100 proteins act through activation of surface receptors in autocrine and paracrine manner [2].

## 2. History

The first mention about “relatively acidic and small in size” proteins appears in the paper written by Moore and McGregor in 1965. Moore and McGregor compared chromatographic patterns of proteins present in the brain and the liver of rabbits and found that brain contained at least two proteins which were in the lower quantity or even absent in the liver [3]. Later on, Rubin and Stenzel described synthesis of the brain protein* in vitro*, which originates in a ribosomal system from the rabbit cortical grey matter. The specific antigen used was an acidic protein, isolated from brain by Rubin and Stenzel [4].

In 1973, Kretsinger and Nockolds described the structure of calcium binding protein, parvalbumin: the main chain consists of six helices, A–F. The loops CD and EF bind the calcium and AB loop does not; nevertheless, its structure is similar to the CD and EF regions, apparently as a result of a gene triplication [5]. Since then, different S100 proteins have been recognized and their expression in various peripheral tissues has been identified [6].

## 3. Structure and Function of S100 Proteins

EF-hand (i.e., helix-loop-helix) superfamily consists of at least forty-five distinct subfamilies. Their members contain from two to eight EF-hands that are recognizable by amino acid sequence [7]. The EF-hand motif consists of two alpha helices “E” and “F” joined by an intervening 12-residue calcium binding loop [8]. The EF-hand motifs are typically arranged in pairs held together by a short antiparallel *β*-strand and numerous hydrophobic interactions between the four helices. S100 family proteins play a specific role among the other members of EF-hand superfamily due to their unique structure and properties [2]:

1. *Dimeric structure of S100 proteins:* S100 proteins exist in a dimeric structure with the exception of S100G, which is monomeric. S100 proteins can exchange their subunits with other members of S100 family to form homodimers and heterodimers in the cellular compartment.
2. *Subunits constitution of S100 proteins:* each subunit (each monomer) is composed of two EF-hand motifs. The first monomer, C-terminal one (“canonical”), is formed by 12 residues and ligates calcium with a higher affinity. The 14-residue, N-terminal (also called “pseudocanonical”) EF-hand binds the calcium with a weaker affinity.
3. *Expression of S100 proteins:* expression of S100 proteins occurs in a tissue- and cell-specific manner [9].

At the low calcium state, S100 proteins present in their calcium-free (apo) state. After the influx of calcium through the voltage-gated or receptor-mediated channels happens, S100 proteins bind calcium and undergo a specific conformational change which leads to a modification of their hydrophobic surface properties [2, 9].

The EF-hand-containing proteins can be functionally divided into two groups. First group consists of calcium sensors, which are characterized by ability to translate the signal of a change in concentration of Ca^2+^ ions to various responses. The second group includes calcium buffers, which bind free Ca^2+^ ions in the cytoplasm of the cell and can modulate calcium signals. Nevertheless, the borderline between these two groups is not strict and many EF-hand proteins can be described neither as clear sensors nor as clear buffers [10].

As mentioned above, increase in calcium levels leads to structural changes of S100 proteins and this allows them to interact with hydrophobic regions of the target proteins. For human S100 family members, more than 90 potential intracellular and extracellular target proteins have been reported: other calcium binding proteins (members of the annexin family), enzymes (e.g., aldolase A/C), cytoskeletal components (actin and tubulin), regulator genes of the cell cycle (p53), second messenger-synthesizing enzymes (adenylate and guanylate cyclase), and kinases belong to the interacting partners [11].

## 4. Gastrointestinal Cancer and S100 Proteins

Carcinogenesis is a multistep process requiring combination of genetic and epigenetic aberrations of normal human cells which lead to their progressive transformation into highly malignant derivatives. In 2000, Hanahan and Weinberg suggested that the malignant growth of nearly all types of cancers is a result of six essential alterations in the cell physiology: self-sufficiency in growth factors, insensitivity to growth-inhibitory signals, evasion of apoptosis, limitless replicative potential, sustained angiogenesis, and the ability to invade and metastasize [12]. In 2011, Hanahan and Weinberg added another two hallmarks of cancer: genomic instability and inflammation. Furthermore, reprogramming of energy metabolism and evading immune destruction have been included as two emerging hallmarks recently [13].

A total of 14.1 million new colorectal cancer cases were diagnosed in 2012 worldwide; there were 32.6 million people surviving with cancer (within 5 years from the diagnosis) and 8.2 million cancer deaths in 2012. According to this colorectal cancer takes prominent place among human malignancies with its third highest incidence in men and second highest incidence in women [14].

A growing interest in S100 proteins has been observed recently, as there is a clear evidence that they are involved in a variety of biological events related to the carcinogenesis. Firstly, majority of S100 genes are found on human chromosome 1q21, which is a region prone to genomic rearrangements. Secondly, altered expression of S100 proteins has been observed in different malignancies. Thirdly, S100 proteins interact with various proteins, which play an important role in carcinogenesis and S100 proteins also exert specific effects on target proteins such as NF-*κβ*, p53, and *β*-catenin [15].

Association of S100 proteins with different types of gastrointestinal cancer is shown in Table 1.

## 5. Colorectal Neoplasia and S100 Proteins

### 5.1. S100A8/A9: Calprotectin

S100A8 and S100A9 are also known as calgranulins A and B or MRP8 and MRP14. They are expressed in a variety of the cells, being abundant especially in the myeloid cells including granulocytes, monocytes, and myeloid-derived suppressor cells, MDSC [16]. Heterodimerization with S100A8 stabilizes S100A9 and causes elongation of its C-terminal *α*-helix. The tetramers create two high affinity Zn^2+^-binding sites, which are important for the function of calprotectin including its antimicrobial activity [2, 16–18]. Calprotectin, heterocomplex of S100A8/A9, plays a key role in the regulation of different inflammatory processes and immune responses. After the neutrophil activation and/or endothelial adhesion of monocytes, calprotectin starts to be secreted and can serve as a marker of inflammation [17]. On the other hand, calprotectin is chemotactic for neutrophils [2, 19, 20]. At present, calprotectin is used as a biomarker and its faecal levels are routinely measured to confirm activity of inflammatory bowel disease.

Heterocomplex of S100A8/S100A9 can influence migration of MDSC and may induce tumour cell invasion [21–23].

Effect of calprotectin towards the tumour cells is dependent on the concentration: at high concentrations, heterocomplex of S100A8/9 exerts an apoptotic effect on colon carcinoma cell lines [24], but at the low concentrations, tumour cell growth is promoted by calprotectin [25]. Low concentrations of S100A8/9 stimulate tumour cell migration [26].

Another important role of calprotectin in the context of increased risk of colorectal cancer in patients with inflammatory bowel diseases was described by Turovskaya et al.: in human colon tumour tissue, myeloid progenitors infiltrate regions of dysplasia and adenoma and express S100A8 and S100A9. RAGE (receptor for advanced glycation end products) is the principal receptor of S100A8/A9 on the surface of tumour cells. Binding of calprotectin to RAGE activates the NF-*κβ* signaling pathway, which plays a key role in the molecular pathogenesis of colitis-associated carcinoma [27]. Ichikawa et al. confirmed that RAGE and carboxylated glycan-dependent binding of S100A8/9 promotes MAPK (mitogen-activated protein kinases) and NF-*κβ* signaling [26].

Recently Lehmann et al. have shown that majority of colorectal cancer patients had increased levels of faecal calprotectin before the surgical tumour resection, which was followed by a significant decrease 3 months after the operation. Patients with T3 and T4 tumors had significantly higher calprotectin levels compared to those with T1 and T2 cancers [28].

### 5.2. S100A4: Metastatin-1, Calvasculin

S100A4, a multifunctional protein localized in the nucleus, cytoplasm, and extracellular space, is strongly associated with metastatic tumour progression [29]. Metastatin-1 was firstly described in 1989 by Ebralidze et al. [30] who found it expressed specifically in metastatic cells. Later on, S100A4 was identified to be a precious biomarker for identification of patients who have already developed metastases or who are at high risk to develop metastases metachronously. Therefore, metastatin-1 is a prognostic biomarker, too. Its role in prediction of tumour progression and prognosis of CRC was confirmed in a meta-analysis performed by Liu et al. [31]. Boye et al. emphasized that the nuclear expression (not the cytoplasmic one) of S100A4 is a novel prognostic marker for colorectal cancer. They also found that the prognostic impact was largely confined to TNM stage II, and patients with stage II tumours expressing nuclear S100A4 had similar prognosis when compared to stage III patients [32]. Recent research focused on calvasculin has shown that S100A4 is not only a biomarker, but it also plays a causative role in mediating metastatic processes [33].

Intracellular S100A4 increases cell motility via interactions with the proteins of cytoskeleton, such as actin filaments, nonmuscle tropomyosin, and nonmuscle myosin II [2, 33]. Furthermore, metastatin-1 induces invasiveness of primary tumors through the interaction with liprin beta 1 which leads to modulation of cell adhesion causing a migratory phenotype [34]. S100A4 contributes to the development of more aggressive cell phenotype via binding to p53 which results in a modulation of p53 transcriptional activity [35].

S100A4, when released into the extracellular space, enables angiogenesis through stimulation of endothelial cells motility and through activation of matrix metalloproteinases expression, which cleave proteins of the extracellular matrix and thereby facilitates cell invasion into the adjacent tissues [29, 33]. Extracellular angiogenic effect of S100A4 is also induced through its interaction with annexin II on the surface of endothelial cells [36].

APC gene mutation, which is present in about 90% of colorectal cancers, leads to an increase of cytoplasmic *β*-catenin, resulting in its nuclear accumulation and expression of genes involved in the tumour growth and invasion [37, 38]. Sack and Stein described that S100A4 is a transcriptional target of *β*-catenin. Therefore, targeting the Wnt/*β*-catenin pathway with the subsequent inhibition of S100A4 gene expression will restrict formation of colon cancer metastases [33]. Calcimycin was shown to inhibit Wnt/*β*-catenin pathway activity and the expression of *β*-catenin target gene, S100A4 [39]. Stein et al. demonstrated the effect of sulindac on colon cancer cell lines: reduced expression of *β*-catenin, followed by decrease of its nuclear accumulation, resulted in knockdown of S100A4 expression. In mice, sulindac treatment reduced tumour growth and decreased liver metastases in a human colon cancer xenograft model [40].

### 5.3. S100A6: Calcyclin

Calcyclin, originally identified in Ehrlich ascites tumour cells, was the first S100 protein described to be related to the cellular proliferation [61, 62]. Indeed, S100A6 is preferentially expressed in proliferating cells when compared to the quiescent ones. Similar to S100A4, calcyclin is associated with the tumour progression and invasion. Komatsu et al. found in their study that expression of S100A6 in human colorectal adenocarcinomas was significantly higher (2.4-fold) when compared to the expression of S100A6 in the normal mucosa. They also demonstrated that positivity of S100A6 antibody was present in 5% of normal mucosa, 46% of adenoma specimens, 55% of adenocarcinomas, and 100% of carcinoma cells that metastasized to the liver. A significant association between Duke's tumour stage, lymphatic invasion, and S100A6 expression was confirmed [41]. Stulík et al. demonstrated statistically significant correlation between S100A6 expression and colon carcinoma progression, too. They investigated S100A6 in normal colon tissue and in colorectal adenomas and carcinomas. They found out that S100A6 accumulates in the invasive margin of the colorectal cancer [62]. Several mechanisms of calcyclin involvement in colorectal carcinogenesis have been documented: Wnt/*β*-catenin pathway and *β*-catenin itself can regulate S100A6 gene expression. Colocalization of calcyclin with *β*-catenin in colorectal cancer tissues has been identified. S100A6 interacts with a protein called lamin A/C, which is implicated in colon carcinogenesis [42]. Furthermore, S100A6 interacts with the receptor for advanced glycation end products and modulates cell survival [63]. Duan et al. studied the contribution of S100A6 to the colorectal carcinogenesis and demonstrated the effect of calcyclin on cell proliferation and migration via mitogen-activated protein kinase activation* in vitro* and tumour growth* in vivo*. S100A6 induced proliferation was partially suppressed by an extracellular receptor kinase inhibitor; migration was attenuated by a p38 mitogen-activated protein kinase inhibitor [64].

Melle et al. demonstrated that S100A6 was significantly upregulated in colorectal cancer and metastases derived from colorectal cancer when compared to hepatocellular carcinoma. This conclusion seems to be very auspicious for clinical practice when the primary tumour is not known [65].

### 5.4. S100A11: Calgizzarin, S100C

Calgizzarin is localised in the cytoplasm and, in contrast to the majority of S100 proteins, in the nucleus of the cell, too [66]. Loss of nuclear calgizzarin in HeLa cells was described to lead to loss of inhibition of the cell growth [67]. S100A11 is involved in regulation of cell motility, cell cycle progression, cell differentiation, transcription, apoptosis, and inflammation [43, 66].

Calgizzarin has been identified to be a ligand of receptor for advanced glycation end products [68]. The role of S100A11 is complex and it can act either as a tumour suppressor or as a tumour promoter in different types of tumours [66]. Melle et al. were looking at expression of S100A11 in normal colonic epithelium, adenoma, and colorectal carcinoma and found upregulation of calgizzarin in colorectal cancer when compared to adenoma and to epithelial cells [43]. Stulík et al. also documented possible participation of calgizzarin in the process of colon cancer development [69].

### 5.5. S100B

S100B was found to be overexpressed in the liver metastases of colorectal cancer patients [70]. Furthermore, Huang et al. tried to detect circulating tumour cells in CRC patients, as the undetected micrometastases play an important role in the early relapse of CRC. They demonstrated that the postoperative relapse was significantly correlated with the gene overexpression including S100B. Based on this finding, S100B might be one of the prognostic markers for CRC patients [44].

### 5.6. S100P

S100P was discovered in 1992 [45, 71]. Ever since it has been proven that S100P is able to promote tumour invasion and formation of metastases [72, 73]. Upregulation of S100P was described in colorectal adenoma when compared to the normal tissue [46]. S100P is significantly upregulated by prostaglandin E2, which is usually overexpressed in epithelial CRC cells [47]. The first proof that the S100P expression in cancer tissues, as well as the serum S100P levels, might be used as a potential prognostic biomarker for CRC patients was documented by Wang et al. Results of their study confirmed significantly higher expression of S100P protein in cancerous tissues compared to the paired noncancerous regions. Interestingly, expression of S100P protein was associated with the localisation of the primary CRC tumour: the rate of S100P expression increased from the right to the left, being the highest in the rectal carcinomas. According to Kaplan-Meier analysis, the overall survival time of patients with CRC staged I–III with S100P protein expression was significantly shorter when compared to those without the expression of S100P protein in the tumour tissue. Survival time of CRC patients was also significantly associated with the preoperative S100P serum levels, investigated by ELISA [74].

## 6. Possible Therapeutical Implications of S100 Proteins

Data show that the inhibition of S100 proteins might play a key role in overcoming resistant forms of different malignancies. Tasquinimod, a novel oral quinoline-3-carboxamide derivative, was shown to have multiple effects on the tumour microenvironment [75] including possible binding to S100A9 protein which leads to the inhibition of interaction between S100A9 and receptors such as RAGE and TLR4. This might have an implication in the therapy of prostate cancer [76].

Dong et al. found that S100P (which is usually overexpressed in the colorectal cancer tissue) decreases chemosensitivity to 5-fluorouracil* in vitro* [73]. Therefore, S100P protein does not have a role of a prognostic indicator only, but also serves as a therapeutic target.

S100A6 protein became a potential prognostic biomarker and also a precious therapeutic target in the study carried out by Zhang et al. [55]: its inhibition decreased the metastatic potential of gastric cancer cells.

At this point we believe that incorporation of S100 proteins/antibodies aimed at S100 proteins will improve the anticancer therapy: it will make it more tailored, will decrease its side effects, and will help in the fight with resistant forms of cancer.

## 7. Conclusions

S100 proteins, members of EF-hand family, play an important role in the colorectal carcinogenesis.

According to the available data, S100 proteins could be used for diagnostic, prognostic, and surveillance purposes in patients who are at risk of or have already been diagnosed with colorectal neoplasia.

Possibly, in the very near future, S100 proteins might be a therapeutic target for tailored anticancer therapy.

## Figures and Tables

**Table 1:** Association of S100 proteins with different types of gastrointestinal cancer.

Cancer site	Type of S100 protein	S100 regulated features	References
Colon	S100A8/A9, S100A4, S100A6, S100A11, S100B, S100P	↑	[25–28, 31–33, 41–47]

Oesophagus	S100A2	↓ in ESCC ↑ in adenocarcinoma	[48, 49]
	S100A9	↑ in adenocarcinoma	[50]

Stomach	S100A2, S100A14	↓	[51, 52]
	S100A2, S100A3, S100A4, S100A6–S100A10	↑	[53–57]

Pancreas	S100A2	Dysregulated	[58]
	S100A4, S100P	↑	[59]
	S100A11	↑ in early stage, ↓ in tumour progression	[60]

ESCC, oesophageal squamous cell carcinoma; ↑, upregulated; ↓, downregulated.
